# Atypical developmental trajectory of local spontaneous brain activity in autism spectrum disorder

**DOI:** 10.1038/srep39822

**Published:** 2017-01-06

**Authors:** Xiaonan Guo, Heng Chen, Zhiliang Long, Xujun Duan, Youxue Zhang, Huafu Chen

**Affiliations:** 1 Center for Information in BioMedicine, Key laboratory for NeuroInformation of Ministry of Education, School of Life Science and Technology, University of Electronic Science and Technology of China, Chengdu 610054, PR China

## Abstract

Autism spectrum disorder (ASD) is marked by atypical trajectory of brain maturation, yet the developmental abnormalities in brain function remain unclear. The current study examined the effect of age on amplitude of low-frequency fluctuations (ALFF) in ASD and typical controls (TC) using a cross-sectional design. We classified all the participants into three age cohorts: child (<11 years, 18ASD/20TC), adolescent (11–18 years, 28ASD/26TC) and adult (≥18 years, 18ASD/18TC). Two-way analysis of variance (ANOVA) was performed to ascertain main effects and interaction effects on whole brain ALFF maps. Results exhibited significant main effect of diagnosis in ASD with decreased ALFF in the right precuneus and left middle occipital gyrus during all developmental stages. Significant diagnosis-by-age interaction was observed in the medial prefrontal cortex (mPFC) with ALFF lowered in autistic children but highered in autistic adolescents and adults. Specifically, remarkable quadratic change of ALFF with increasing age in mPFC presented in TC group was absent in ASD. Additionally, abnormal ALFF values in diagnosis-related brain regions predicted the social deficits in ASD. Our findings indicated aberrant developmental patterns of spontaneous brain activity associated with social deficits in ASD and highlight the crucial role of the default mode network in the development of disease.

Autism spectrum disorder (ASD) is a prototypically early-onset neurodevelopmental disorder characterized by persistent deficits in social interaction and communication and restricted, repetitive patterns of behavior, interests or activities^1^. The prevalence of ASD was reported to be 1 in 68 children with a rising tendency^2^. Though the specific etiology of ASD remains elusive, there is consensus that the spontaneous brain activity is disturbed in individuals with ASD^3^,^4^,^5^.

Emerging evidence supports that ASD undergoes an atypical trajectory of brain maturation that probably affect autistic symptoms across the lifespan^6^. Previous longitudinal and cross-sectional magnetic resonance imaging (MRI) studies reported age-specific anatomical abnormalities in ASD, which proposed an overgrowth in early life but an accelerated decline during adolescence and young adulthood^7^. Specifically, the frontal lobe showed the most severe enlargement in ASD beginning between 2 and 3 years of age and the frontal grey matter developed at an atypical growth rate in children with ASD^8^,^9^. Cortical thickness studies also clarified abnormal longitudinal neurodevelopmental trends with regional specificity in individuals with ASD, which suggest that the cortical development in ASD first undergoes an expansion at a high rate in early childhood, then accelerated thinning until adolescence, and finally slow down the speed of thinning in early adulthood^10^. In addition, functional connectivity studies identified age-related changes on functional connectivity in ASD. Previous cross-sectional functional connectivity study reported that individuals with ASD exhibited atypical developmental trajectory of default mode network (DMN) connectivity across childhood and adolescence and significant interaction between diagnosis and age was observed in several DMN regions, such as the medial prefrontal cortex (mPFC)^11^. Functional connectivity circuits of the posterior superior temporal sulcus has also been shown to exhibit atypical developmental trajectories in ASD^12^. Research examining developmental changes in large-scale network functional connectivity demonstrated that ASD exhibited different abnormalities patterns of within- and between-network connectivity during different developmental stages^13^. A review of functional connectivity literature put forward a developmental model to account for the age-specific over- and under-connectivity findings in ASD, i.e. hyper-connectivity in children while hypo-connectivity in adolescents and adults^14^. All these findings suggest atypical cortical developmental trajectories across the lifespan and highlight the importance of taking different developmental stages into account when exploring the potential neural mechanisms in ASD.

In recent years, resting-state functional magnetic resonance imaging (rsfMRI), which examines the spontaneous low-frequency fluctuations (LFF) in blood oxygenation level dependent (BOLD) signals^15^, has emerged as a new avenue to explore the pathophysiology underlying neurologic and psychiatric diseases^16^,^17^. LFF has been validated to reflect the spontaneous neural activity (SNA)^18^,^19^ and has been consistently reported to be correlated with electroneurophysiological activity, such as local filed potentials^18^,^20^, indicating that LFF might serve as a meaningful indicator for SNA in the brain^21^,^22^. Additionally, the amplitude of LFF (ALFF) might also be used to assess the intensity of regional SNA, as well as cerebral physiological states^23^,^24^,^25^. In view of its temporal stability^26^ and test-retest reliability^27^, ALFF has been suggested as a powerful index for assessing changes in SNA that are associated with neuropsychiatric disorders^23^,^28^,^29^. Moreover, ALFF exhibited remarkable ability in uncovering the age-related variation in intrinsic brain activity during healthy aging^30^,^31^. Age-specific changes in ALFF were mostly observed in medial wall structures in healthy adults^31^. Specifically, decreased ALFF with increasing age in the posterior cingulate cortex was consistently reported in mature human brain during healthy aging^30^,^31^. These findings implicated the potential of ALFF to investigate developmental changes of baseline SNA in the brain.

Building on the physiopathological significance of ALFF, this study aimed to examine ASD-related regional spontaneous brain activity abnormalities in resting-state from a developmental perspective using ALFF measure. We explored the differences in whole brain ALFF between ASD and typical controls (TC) across three age groups: child (<11 years), adolescent (11–18 years) and adult (≥18 years). In addition, we sought to depict the abnormal developmental trajectory from childhood to adulthood in ASD. Following plentiful studies showing the atypical frontal development in ASD^7^,^9^,^10^,^32^, we predict that the abnormal age-related changes of ALFF in individuals with ASD would probably exist in the frontal cortex.

## Results

### Significant main effects and interaction effects

**Figure 1:**
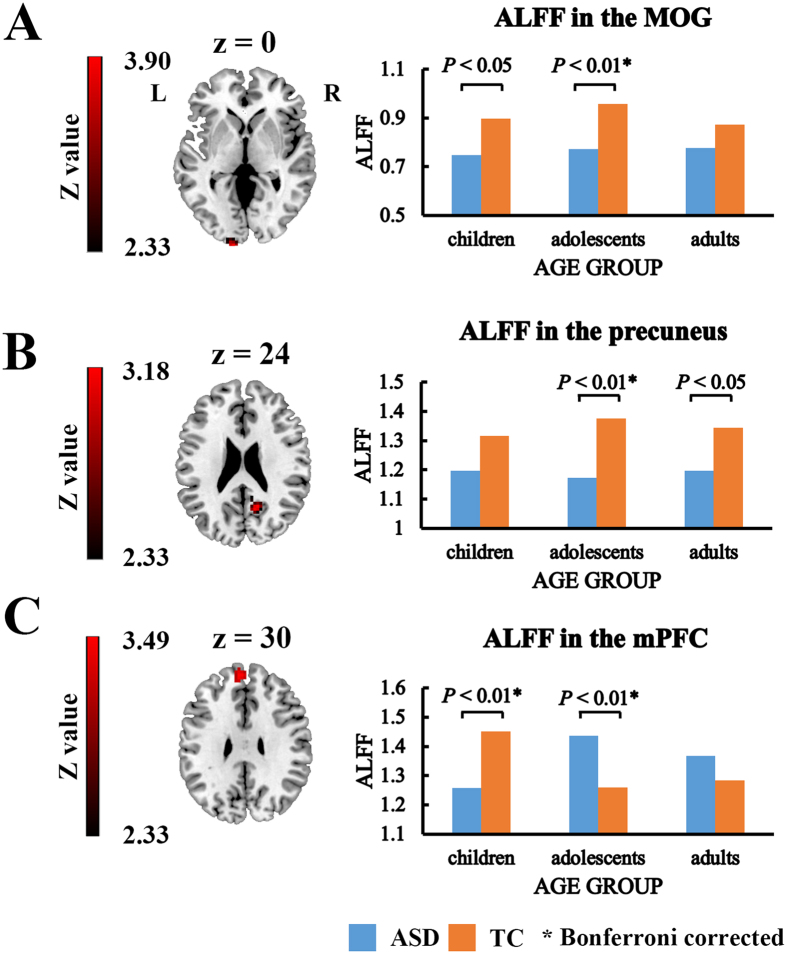
Significant diagnosis-related effects. (**A**) Significant main effect of diagnosis on ALFF in the left middle occipital gyrus. (**B**) Significant main effect of diagnosis on ALFF in the right precuneus. (**C**) Significant diagnosis-by-age interaction effect in the mPFC. mPFC: medial prefrontal cortex; MOG: Middle occipital gyrus; ASD: autism spectrum disorder; TC: typical controls; *indicates Bonferroni corrected.

The whole brain analysis of variance (ANOVA) results exhibited significant diagnosis-related effects, namely main effect of diagnosis and diagnosis-by-age interaction effect (Fig. 1). Significant main effect of age was also observed in the analysis on ALFF (Supplementary Material and Figure S1).

### Main effect of diagnosis

The ANOVA analysis on ALFF demonstrated a significant main effect of diagnosis in the left middle occipital gyrus (partial η^2^ = 0.102; *F*_(1,119)_ = 13.54; *p* < 0.001) and right precuneus (partial η^2^ = 0.097; *F*_(1,119)_ = 12.82; *p* < 0.001) (Gaussian random field (GRF) correction, *Z* > 2.3 combined with cluster size >38 voxels) (Fig. 1A,B and Table 1). Post-hoc analysis showed that ASD group exhibited significantly decreased ALFF values in both regions when compared with TC groups irrespective of age (*p* < 0.001). Specifically, comparisons within the age group showed that the decrease in ALFF was significant merely in adolescent (*p* < 0.01, Bonferroni corrected) and adult (*p* < 0.05, uncorrected) in the right precuneus, as well as in child (*p* < 0.05, uncorrected) and adolescent (*p* < 0.01, Bonferroni corrected) in the left middle occipital gyrus (*p* < 0.05) (Fig. 1A and B).

### Diagnosis-by-age interaction

Significant interaction effect was observed in bilateral mPFC (partial η^2^ = 0.132; *F*_(2,119)_ = 9.03; *p* < 0.001) (GRF correction, *Z* > 2.3 combined with cluster size >51 voxels) (Fig. 1C and Table 1). The ALFF was significantly decreased in children with ASD (*p* < 0.01, Bonferroni corrected), significantly increased in adolescent with ASD (*p* < 0.01, Bonferroni corrected), and showed an increase tendency in adults with ASD (Fig. 1C).

### Modeling ALFF as a function of age

**Figure 2:**
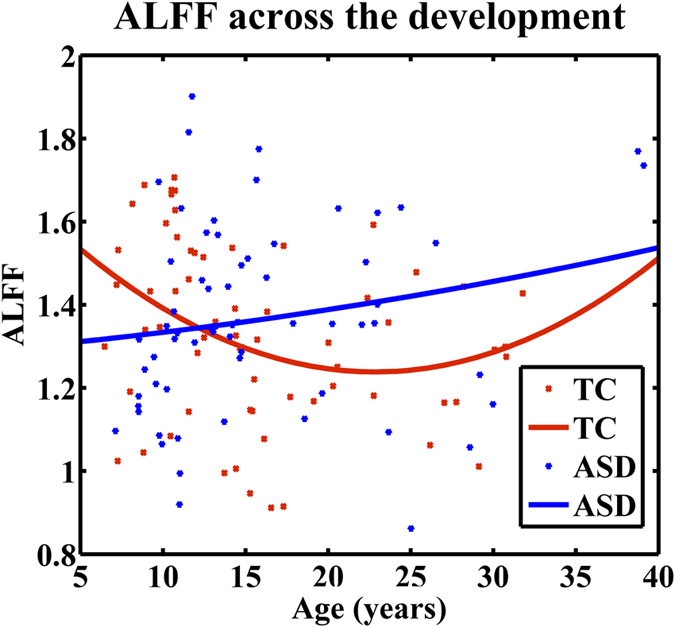
Quadratic correlation between ALFF and age in the mPFC. In the TC group, ALFF in the mPFC changed as a quadratic function of age (*p* = 0.036). In individuals with ASD, ALFF in the mPFC didn’t change as a second-order function of age (*p* = 0.311). ASD: autism spectrum disorder; TC: typical controls.

Since the ANOVA results showed that diagnosis interacted with age group to affect ALFF in mPFC, we directly examined the ALFF values in mPFC as a quadratic function of age for both diagnosis groups (Fig. 2). Results revealed that remarkable quadratic change in ALFF with increasing age presented in TC group (p = 0.036) was absent in ASD (p = 0.311).

### Abnormal ALFF values predicts symptom severity in the ASD group

We investigated the relationship between ALFF values in brain regions exhibiting significant diagnosis-related effects and ADOS subscale scores using multivariate regression model and leave-one-out cross validation (LOOCV) procedure. Non-parametric permutation test was performed to assess the statistical significance. Results showed that ALFF in the mPFC, precuneus and middle occipital gyrus predicted the social subscore of ADOS in the ASD group (*p* = 0.005, Bonferroni corrected). However, regression analysis predicting scores on communication or restricted and repetitive behaviors domains of ADOS didn’t yield significant correlations.

## Discussion

In the current study, we examined the age effect on spontaneous brain activity reflected by ALFF in ASD and TC. Compared with TC group, ASD group exhibited significant lower ALFF values in the right precuneus, a key node of posterior default mode network (DMN), and left middle occipital gyrus across three age groups. In contrast, the mPFC, a key node of anterior DMN, showed a significant diagnosis-by-age interaction effect. We further found that autistic children and adolescents exhibited significantly decreased and increased ALFF respectively, when compared with typically developing peers. Specifically, the developmental trajectory of ALFF in ASD is apparently different from that in TC. Aberrant spontaneous brain activity in diagnosis-related brain regions also linked with the severe impairments in the social domain in ASD across the lifespan. Our findings demonstrates aberrant developmental patterns of spontaneous brain activity associated with social deficits in individuals with ASD and highlight the potential importance of DMN in probing into the pathophysiology of ASD.

The DMN is a set of distributed, functionally connected cortical nodes including the mPFC, posterior cingulated cortex (PCC) and precuneus^33^, and involved in Theory of Mind^34^, social and self-referential cognitive processes^35^. The DMN has been identified as a network active in the resting-state brain while attenuated during goal-directed cognitive tasks^36^. Increasing evidence suggested atypical DMN anatomy and function in individuals with ASD^36^,^37^,^38^,^39^.

As we expected, compared with TC, individuals with ASD exhibited apparently different developmental trajectory in the mPFC. In addition, compared with TC group, ASD exhibited distinct pattern in the mPFC through childhood to adulthood (i.e. lower in the children, higher in the adolescents and similar in adults). Developmental alterations of either functional connectivity or metabolism in the mPFC have been frequently observed in individuals with ASD^32^,^37^,^40^. Our results are consistent with those previous findings associated with developmental functional abnormalities in the mPFC, and reiterating the involvement of the mPFC in the development of ASD. As we mentioned in the introduction, previous developmental studies from the perspective of grey matter volume and cortical thickness indicated that the anatomic abnormalities in ASD have the age-specificity^7^,^10^. At the same time, the amplitude of BOLD fluctuations in brain regions revealed by ALFF might partly reflect the local electrophysiological activity^4^,^18^,^20^. Age-dependent structural abnormalities might explain the atypical brain activity during the development of the ASD group in the mPFC. Furthermore, it seems appropriate to conclude that dysfunction in the mPFC might be a significant feature of ASD across the life span.

The mPFC has been associated with mentalizing^41^ and self-referential mental activity^35^. Motivated by the social cognitive impairments in ASD, several studies have investigated the social cognitive function of mPFC in individuals with ASD^38^,^42^,^43^. Individuals with ASD displayed reduced activation in the mPFC while making true/false judgments for self- and other-reflection statements^42^. In another mentalizing task, ASD exhibited reduced activation in the mPFC in relative to control group^43^. In addition, typical controls activated the mPFC more for self-referential than other-referential processing, while ASD activated equally to self and other^38^. Taking account of these findings, we speculate that the dysfunction of the mPFC might underlie the social and Theory of Mind impairments in ASD^38^.

The precuneus plays a crucial role in high-level cognition functions, including episodic memory, consciousness and self-referential cognitive processes^35^,^44^,^45^,^46^. Numerous neuroimaging studies have provided support for the precuneus abnormalities in ASD from either anatomy or function^47^,^48^,^49^. Atypical hyperactivation during the social moral judgments task in the precuneus highlights the dysfunction of social information processing in ASD^48^. Moreover, individuals with ASD also exhibited increased activity in the precuneus when processing facial emotions^50^. The reduced ALFF at rest might provide an alternative explanation for failure to deactivation during cognitively demanding tasks^51^. Atypical ALFF found in our study suggests that abnormal mental process might exist in the precuneus in ASD and indicates that the social and emotional deficits in ASD might be associated with the dysfunction of the precuneus^51^.

Notably, the anterior (i.e. mPFC) and posterior (i.e. precuneus) nodes of the DMN in ASD exhibited differential abnormalities patterns under the influence of age. The mPFC showed age-specific abnormalities while the precuneus showed decrease among all the age groups. DMN-related functional connectivity abnormalities in individuals with ASD have been confirmed in abundant resting-state studies^37^,^47^. It is possible that these distinct developmental effects on ALFF in ASD are connected to the disruption in coordination between the anterior and posterior DMN during the maturation in ASD^37^. Moreover, subjects with ASD exhibited significantly altered ALFF in the mPFC in childhood, which is much earlier than the existence of abnormalities in the precuneus. The prefrontal cortex is one of the last regions to mature and its function is sensitive to changes with age, and the earlier alteration in this region might be attributed to that brain regions, which are most plastic during the development, are more tend to suffer from the external factors^52^. Overall, our findings provide new evidence for the DMN-related developmental abnormalities in individuals with ASD, and highlight the potential role of DMN in the pathological mechanism underlying ASD.

In the current study, the left middle occipital gyrus exhibited significantly decreased ALFF values in ASD compared with TC group, which might partly be ascribed to reduced level of metabolism in the occipital gyrus in individuals with ASD^53^. Particularly, reduced spontaneous brain activity exists across the lifespan in individuals with ASD. The results replicated the previous study^54^, which also found decreased low-frequency oscillations in this region. An increasing number of studies have demonstrated that individuals with ASD showed atypical activation in the middle occipital gyrus during the visual processing tasks^55^,^56^. Additionally, individuals with ASD exhibited higher activation in visual-perceptual regions together with lower activation in high-order or social-related brain regions during social tasks, albeit without behavioral differences^57^, which indicates unique neural systems underlying ASD for social stimuli. For example, ASD activated the occipital gyrus abnormally instead of the traditional fusiform face area during the face processing task^58^. Therefore, it seems possible to conclude that the dysfunctional localized brain activity in the middle occipital gyrus might be involved in the social deficits in individuals with ASD.

Our multivariate regression analysis results further revealed direct associations between aberrant ALFF values and ASD symptoms and highlighted the significance of spontaneous brain activity in uncovering the pathophysiology of ASD. ALFF in the DMN and middle occipital gyrus predicted the complex behavioral manifestations in social domain in ASD, which indicated that abnormal spontaneous brain activity might underlie social deficits in ASD. ASD is characterized by prominent impairments in social behaviors, such as poor eye contact, lack of social or emotional reciprocity, impairment in the use of nonverbal behaviors and failure to develop peer relationships^1^. These social cognitive deficits have been linked with a diverse range of brain regions, most of which consistently reported the brain function abnormalities in DMN^36^,^37^,^38^,^39^. The relationship between the DMN and social domain deficits might partly be attributed to the special role of DMN in social and self-referential cognition^35^. It seems that dysfunction within the DMN might lead to abnormal self-referential process and could further result in atypical social behavioral characteristics in ASD. The brain-behavior relationship found in the current study might provide additional evidence for understanding the pathologic mechanisms underlying core symptoms in individuals with ASD.

Previous rsfMRI studies examining ALFF in ASD^3^,^4^,^54^, despite highlighting the potential importance of spontaneous brain activity in ASD, either focused on a single age group or mixed age groups without measuring the impact of development, and none of them has examined the regional spontaneous brain activity revealed by ALFF in a developmental context in ASD. In the current study, individuals with ASD were found to have decreased ALFF in the right precuneus and left middle occipital gyrus during three developmental stages compared with age-matched control groups. Similar results were also reported in previous publication using ABIDE database, where they found reduced ALFF in the left middle occipital gyrus in individuals with ASD for all ages^54^. While they also reported ASD-related increases in ALFF in the right dorsal superior frontal cortex, which was not found in our main effect of diagnosis results. Previous ALFF study in adults with high-functioning ASD reported inconsistent findings that individuals with ASD exhibited reduced ALFF in the right occipital gyrus, lingual gyrus and fusiform gyrus^4^. Another study reported higher whole brain ALFF values in children with ASD^3^. To the best of our knowledge, only one study examined age-specific effects on ALFF in ASD, which focused on ALFF in the posterior superior temporal sulcus^12^. However, no significant between-group difference was found for ALFF and no significant diagnosis-by-age interaction effect was found in the posterior superior temporal sulcus, which suggested that the spontaneous brain activity reflected by ALFF was not affected by ASD in the posterior superior temporal sulcus.

Dissimilarities between previous findings and the current study in ASD might partly be attributed to several factors. Since the current study specifically focused on investigating the developmental effects on spontaneous brain activity revealed by ALFF in individuals with ASD, we included one dataset which spanned a large age range, thus variation in results might be related to inter-scanner variability in multicenter studies (e.g., scanner sequences)^59^,^60^. In addition, previous ABIDE analysis included a wider range of subjects^54^, which might also explained the inconsistent findings. At the same time, ASD has been increasingly recognized as a spectrum disorder, and varies greatly depending on the symptom severity, developmental level and age^1^. Hence the sample characteristics resulting from heterogeneity of symptomatology in ASD might lead to the different findings. Further, some differences in methodology could also explain the discrepancies, such as standardization procedure.

## Conclusion

In the current study, we sought to explore how ALFF change with age in individuals with ASD and TC. Atypical ALFF values were found in the precuneus and occipital gyrus. In addition, the anterior and posterior DMN showed different abnormal patterns and onset time of abnormal brain activity in ASD. During typical development, ALFF in the mPFC is higher in children than in adolescents and adults. In contrast, the developmental trajectory of ASD group in the mPFC is non-significant. Symptom severity prediction analysis showed that abnormal ALFF values in these three regions predicted the social impairments in ASD. Our findings provide new evidence for the developmental abnormalities of low-frequency oscillations in ASD, which might underlie the ASD impairments in social domain and highlight the crucial role of the DMN in the development of the disease.

## Methods

### Subjects

We utilized the rsfMRI and phenotypic data collected at the New York University (NYU) Langone Medical Center from the open-access Autism Brain Imaging Data Exchange database (ABIDE, http://fcon_1000.projects.nitrc.org/indi/abide/)^54^ in this study. Only NYU dataset was utilized in the current study for several reasons. First, NYU dataset spans a large age range (6 years to 40 years), which is appropriate for neurodevelopmental studies. Second, it includes participants of three developmental stages (i.e., child, adolescence and adult), and each diagnostic group within each age group has more than 10 subjects after excessive head motion exclusion and diagnostic group matching procedure. Third, since no other dataset satisfied the above requirements, only NYU dataset was included in the analysis to avoid cross-study variability.

ASD participants included in the present study had a clinical Diagnostic and Statistical Manual of Mental Disorders, Fourth Edition, a text revision (DSM-IV-TR) diagnosis of Autistic Disorder, Asperger’s Disorder, or Pervasive Developmental Disorder Not-Otherwise-Specified using the Autism Diagnostic Observation Schedule (ADOS)^61^, and when possible, the Autism Diagnostic Interview Revised (ADI-R)^62^. Estimates of the full-scale intelligence quotient (FIQ) were obtained based on the Wechsler Abbreviated Scale of Intelligence (WASI)^63^. Originally there are 184 subjects in total. 7 subjects with excessive head motion were excluded (i.e. translational or rotational head motion exceeded 3 mm or 3°). The age, gender, FIQ and mean frame-wise displacement (FD)^64^ of ASD and TC participants were matched within the age group (Table 2). Since the current study aimed to explore the effects of age on ALFF in ASD and TC individuals, ages of all participants were restricted to be within 3 standard deviations (SD) from the mean age in each age group for standardization. 49 subjects were not included based on the matching procedure. Finally, 128 subjects (64 ASD and 64 TC) were included in the current study. All the participants were further classified into child (<11 years, 18ASD/20TC), adolescent (11–18 years, 28ASD/26TC) and adult (≥18 years, 18ASD/18TC) groups to explore the effects of age on ALFF. More detailed information on the diagnostic protocols are publicly available at http://fcon_1000.projects.nitrc.org/indi/abide/.

### Ethical statements

All experimental protocols were approved by the NYU institutional review board. The methods were carried out in accordance with the approved guidelines. Written informed consent was obtained from all participants. All of our data are from the open-access dataset ABIDE project. Please find more detailed information on the ethical statements at http://fcon_1000.projects.nitrc.org/indi/abide/.

### Data acquisition

MRI scans were performed on a 3 Tesla Siemens Allegra scanner using an echo-planar imaging (EPI) sequence with a whole-brain coverage (TR/TE = 2000/15 ms, voxel size = 3 × 3 × 4 mm^3^, number of slice = 33, flip angle = 90°, slice thickness = 4 mm, field of view = 240 mm). Each resting-state scan lasted 6 minutes for a total of 180 volumes.

### Data preprocessing

Image preprocessing was conducted using the advanced edition of the Data Processing Assistant for Resting-State fMRI (DPARSF *A*)^65^. Image preprocessing steps included discarding of first ten volumes, slice-timing correction, spatial realignment (participants with translational or rotational motion greater than 3 mm or 3° were excluded), normalization to the standard EPI template in Montreal Neurological Institute (MNI) stereotaxic space and resampling to 3 × 3 × 3 mm^3^, spatial smoothing with an isotropic Gaussian kernel (full width at half maximum = 8 mm), the removal of linear trends, nuisance covariates regression [Friston 24 motion parameters^66^,^67^,^68^, white matter (WM) signal and cerebrospinal fluid (CSF) signal] and band-pass filtering (0.01–0.08 Hz).

### ALFF calculation

In order to obtain the individual whole brain ALFF map, the time series of each voxel was transformed to the frequency domain using a fast Fourier transform (FFT) and the power spectrum was then obtained. The square root of the power spectrum was calculated and then averaged across 0.01–0.08 Hz at each voxel. This averaged square root was regarded as the ALFF^23^. For the purpose of standardization, the ALFF value of each voxel was further divided by the global mean ALFF value to reduce the global effects of variability across subjects^23^.

### Statistical analysis

Two-way ANOVA with diagnosis (two levels: ASD and TC) and age (three levels: child, adolescent and adult) as between-subject factors was performed using statistical parametric mapping (SPM12, http://www.fil.ion.ucl.ac.uk/spm/). The gender, FIQ and mean FD were taken as covariates in the model. The multiple comparisons of each main effect and interaction effect were corrected using Gaussian random field theory implemented in the Resting State fMRI Data Analysis Toolkit (REST, http://www.restfmri.net). The significantly statistical level was accomplished by combining voxel-level threshold of *p* < 0.01 (*Z* > 2.33) and cluster-level threshold of *p* < 0.05. Brain regions showing significant main effect of diagnosis and interaction effect were treated as regions of interest (ROIs). Post-hoc analysis was conducted to compare the strength of ALFF between ASD and TC by random effects two-sample *t*-test, followed by Bonferroni correction (*q* < 0.05, p = 0.05/3 = 0.017).

### Modeling ALFF as a function of age

To further ascertain the specific effects of age on ALFF values in the brain regions showing significant interaction effect, we modelled the ALFF values against age with quadratic function in ASD group and TC group separately. The quadratic fits tend to match the developmental curve of cortical grey matter^69^. *F*-tests were performed to assess the significance level of the models.

### Prediction analysis of symptom severity in the ASD group

We explored whether ALFF in brain regions identified by diagnosis-related effects predicted ASD symptom severity as assessed by ADOS subscale scores in ASD group. Multivariate support vector regression (SVR) approach was utilized to model the relationship between the dependent variable (communication, social, restricted and repetitive behaviors subscore of ADOS) and the multiple independent variables (voxel-wise ALFF values in brain regions showing significant main effects of diagnosis and interaction effects)^70^. The complex symptom manifestations of ASD are associated with the combined effects of multiple spatially distributed brain regions rather than the independent effects of a single region^6^,^37^,^71^,^72^,^73^,^74^. Conventional univariate correlation analyses adopt a single spatial average measure of regional information, which blurs out fine-grained spatial patterns. In contrast, multivariate regression analyses integrate information at each voxel in different brain regions by a simultaneous analysis to predict symptoms. Furthermore, multivariate regression analyses consider the interaction of spatial patterns between multiple features instead of examining voxels in isolation. Thus multivariate regression analyses are more sensitive to decode the relationship between complex brain activity patterns and clinical symptoms in ASD. We used linear kernel support vector machine (SVM) implemented on LibLinear toolbox for regression analysis (http://www.csie.ntu.edu.tw/~cjlin/liblinear/)^75^. A leave-one-out cross validation (LOOCV) procedure was applied to evaluate the performance of regression model^76^. Suppose there are n samples, in each LOOCV trial, the regression model was constructed based on n-1 samples, and then predicted the remaining one. This procedure was repeated n times, and the correlation coefficient value R was calculated between the predicted and observed values subsequently. Finally, the statistical significance was assessed by a non-parametric permutation test^77^. In each trial of permutation test, the actual labels were shuffled randomly, and then the same entire regression procedure was conducted to obtain an R_perm_ value based on the shuffled dataset. This procedure was repeated 1000 times in our study and the final p value was determined by the proportion of the number of times that R_perm_ is larger than the original R in total permutation times (here is 1000). Bonferroni correction was performed for multiple comparisons (*q* < 0.05, p = 0.05/3 = 0.017).

## Additional Information

**How to cite this article:** Guo, X. *et al*. Atypical developmental trajectory of local spontaneous brain activity in autism spectrum disorder. *Sci. Rep.* **7**, 39822; doi: 10.1038/srep39822 (2017).

**Publisher's note:** Springer Nature remains neutral with regard to jurisdictional claims in published maps and institutional affiliations.

## Supplementary Material

Supplementary Information

## Figures and Tables

**Table 1:** Brain regions showing significant diagnosis-related effects.

	Region	Hemi	Voxels	BA	MNI coordinates			*Z* value
					*x*	*y*	*z*	
Main effect of diagnosis								
Cluster 1	Precuneus	R	43	23	15	−57	24	3.18
Cluster 2	MOG	L	39	17/18	−12	−105	0	3.90
Interaction effect								
Cluster 1	mPFC	L	69	9/10	−6	54	30	3.49
	mPFC	R	11	9	3	51	39	3.06

Hemi: hemisphere; L: left; R: right; BA: Brodmann Area; MOG: Middle occipital gyrus; mPFC: medial prefrontal cortex.

**Table 2:** Sample characteristics of the participants.

	ASD (n = 64)	TC (n = 64)	*p*-value
**Children**			
Age (years)	9.60 ± 1.05	9.28 ± 1.45	0.44^a^
Age range (years)	7.13–10.9	6.47–10.86	—
Gender (male/female)	17/1	19/1	0.94^b^
FIQ	110.61 ± 19.94	112.15 ± 12.27	0.77^a^
Mean FD	0.14 ± 0.05	0.13 ± 0.05	0.44^a^
ADOS_COMM	3.11 ± 1.84	—	—
ADOS_SOCIAL	7.28 ± 3.03	—	—
ADOS_STEREO	2.5 ± 1.76	—	—
**Adolescents**			
Age (years)	13.71 ± 1.79	14.46 ± 1.89	0.14^a^
Age range (years)	11.01–17.88	11.56–17.7	—
Gender (male/female)	23/5	21/5	0.90^b^
FIQ	103.57 ± 15.45	104.31 ± 13.51	0.85^a^
Mean FD	0.16 ± 0.08	0.13 ± 0.07	0.22^a^
ADOS_COMM	3.64 ± 1.52	—	—
ADOS_SOCIAL	8.64 ± 2.98	—	—
ADOS_STEREO	2.71 ± 1.44	—	—
**Adults**			
Age (years)	25.41 ± 5.87	25.47 ± 4.16	0.97^a^
Age range (years)	18.58–39.1	19.13–31.78	—
Gender (male/female)	14/4	14/4	1^b^
FIQ	108.06 ± 13.86	110.11 ± 7.87	0.59^a^
Mean FD	0.11 ± 0.04	0.10 ± 0.04	0.73^a^
ADOS_COMM	3.72 ± 1.36	—	—
ADOS_SOCIAL	7.44 ± 3.15	—	—
ADOS_STEREO	1.33 ± 0.91	—	—

^a^Indicates *p* values for two-sample t-test; ^b^indicates *p* values for χ^2^ test. ASD: autism spectrum disorder; TC: typical controls; FIQ: the full-scale intelligence quotient; FD: frame-wise displacement; ADOS_COMM: communication total subscore of the classic ADOS; ADOS_SOCIAL: social total subscore of the classic ADOS; ADOS_STEREO: stereotyped behaviors and restricted interests total subscore of the classic ADOS.
