# Supplementary material for: Atypical developmental trajectory of local spontaneous brain activity in autism spectrum disorder
Source: Sci Rep. 2017 Jan 6;7:39822. doi: 10.1038/srep39822 (PMC5216408; doi:10.1038/srep39822)
Supplement: Supplementary Information [file srep39822-s1.pdf]

Supplementary materials

Atypical developmental trajectory of local spontaneous brain activity in autism spectrum disorder

Running title: Development of spontaneous brain activity in ASD

Xiaonan Guo, Heng Chen, Zhiliang Long, Xujun Duan*, Youxue Zhang, Huafu Chen*

Center for Information in BioMedicine, Key laboratory for NeuroInformation of Ministry of Education, School of Life Science and Technology, University of Electronic Science and Technology of China, Chengdu 610054, PR China.

* Corresponding author: Huafu Chen, Center for Information in BioMedicine, Key laboratory for NeuroInformation of Ministry of Education, School of Life Science and Technology, University of Electronic Science and Technology of China, Chengdu 610054, PR China.

Fax: 86-28-83208238.

E-mails: chenhf@uestc.edu.cn (Huafu Chen), duanxujun@uestc.edu.cn (Xujun Duan).

Methods and results

1. Main effect of age

A distributed set of brain regions showed significant main effect of age, such as the frontal gyrus, cerebellum, thalamus, temporal gyrus, parietal gyrus, angular gyrus and striatum (Gaussian random field (GRF) correction, $Z > 2.3$ combined with cluster size > 88 voxels) (Figure S1 and Table S1).

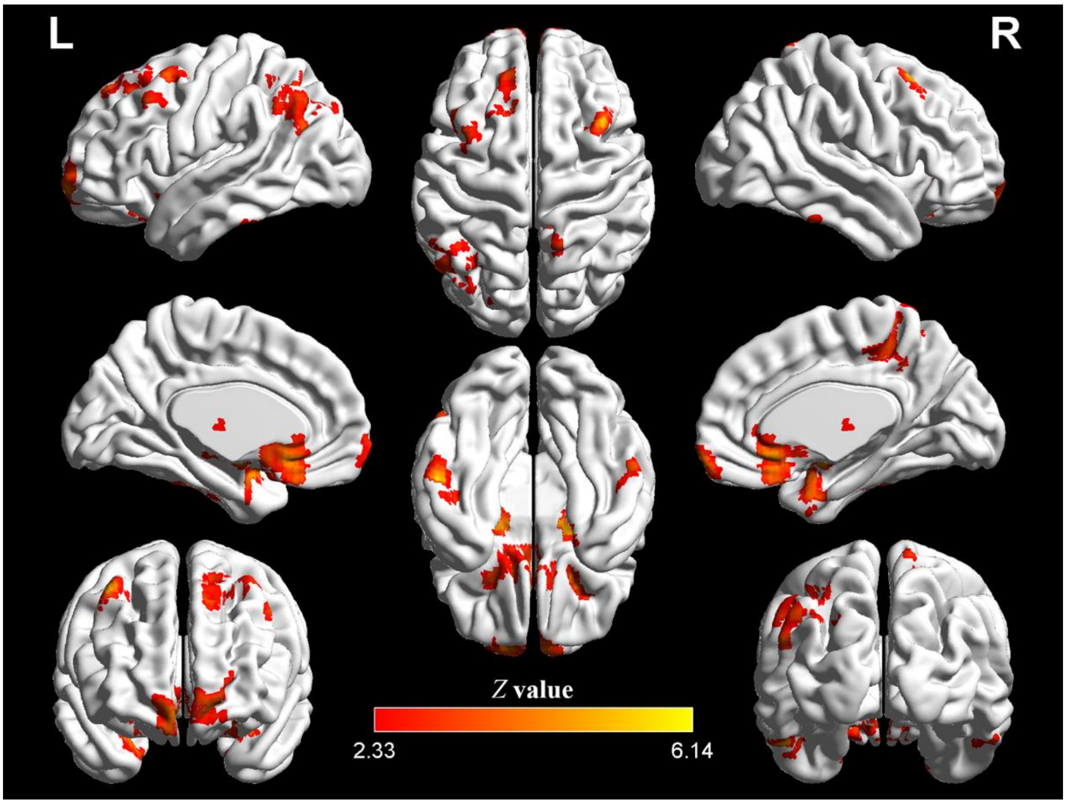

Figure S1 Main effect of age. Brain regions showing significant main effect of age revealed by the two-way ANOVA analysis.

	Region	Hemi	Voxels	BA	MNI coordinates			*Z* value
					x	*y*	*z*	
Cluster 1	Cerebellum	L/R	663	-	21	-72	-39	4.60
Cluster 2	Amygdala	R	121	34	21	-3	-12	5.37
	Parahippocampal gyrus	R		28	21	6	-33	4.22
Cluster 3	Amygdala	L	104	34	-18	-3	-12	5.70
	Hippocampus	L		34	-18	-6	-12	5.60
Cluster 4	Inferior temporal gyrus	L	98	20	-54	-30	-27	5.44
Cluster 5	Striatum	L/R	567	11/25	18	18	-6	6.14
	Gyrus rectus	L/R		11	0	24	-15	4.89
Cluster 6	Orbitofrontal gyrus	L/R	184	11	6	66	-12	4.38
Cluster 7	Thalamus	L/R	202	-	21	-18	0	4.34
Cluster 8	Inferior parietal gyrus	L	219	40	-39	-51	51	4.10
	Angular gyrus	L		39	-45	-72	42	3.83
Cluster 9	Middle frontal gyrus	R	88	8/9	42	18	57	5.82
Cluster 10	Middle frontal gyrus	L	95	6	-36	9	54	4.34
Cluster 11	Precuneus	R	152	5	12	-42	66	3.69
Cluster 12	Superior frontal gyrus	L	109	8/9	-12	42	51	3.68

Table S1 Brain regions showing significant main effect of age. Hemi: hemisphere; L: left; R: right; BA: Brodmann Area.

2. Replication analysis using male subjects

Considering recent reports on differential effects of sex on neurobiology and neurocognitive profiles of ASD[1-3], we conducted additional reproducible analysis excluding female subjects to explore whether the reported neurodevelopmental effects hold for the male subsample. All the selected participants (n = 108) were classified into child (17ASD/19TC), adolescent (23ASD/21TC) and adult (14ASD/14TC) groups. No significant differences of the age, FIQ and mean frame-wise displacement (FD) were found between diagnostic groups within each age group. Two-way analysis of variance (ANOVA) was performed to ascertain main effects and interaction effects on whole brain ALFF maps. FIQ and mean FD were taken as covariates in the model. All the other preprocessing and processing methods remained the same as the main text. The ANOVA analysis demonstrated significant main effects of diagnosis in the right precuneus and left fusiform gyrus (Figure S2 A and B). Significant interaction effect was observed in the bilateral medial prefrontal cortex (mPFC) (Figure S2 C and D). These results indicated that the anterior (i.e. mPFC) and posterior (i.e. precuneus) nodes of the default mode network in ASD exhibited discrepant abnormalities patterns during different developmental stages. This analysis confirmed the robustness of our findings to sexual dimorphism.

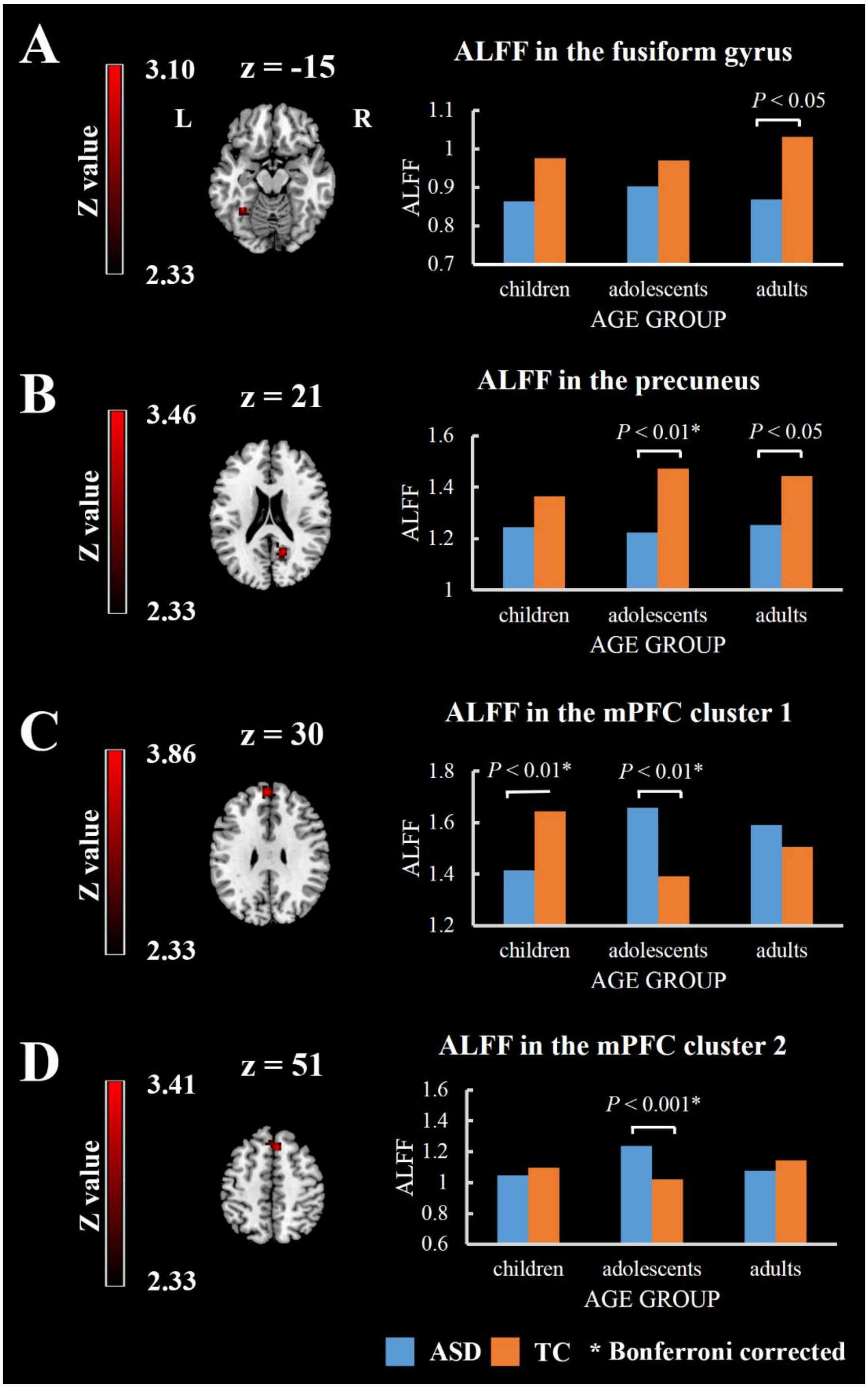

Figure S2 Significant diagnosis-related effects using male subjects. Significant main effect of diagnosis on ALFF in the left fusiform gyrus (A) and right precuneus (B). Significant diagnosis-by-age interaction effect in the mPFC (C and D). mPFC: medial prefrontal cortex; ASD: autism spectrum disorder; TC: typical controls; * indicates Bonferroni corrected.

3. Robustness of findings to head motion

To demonstrate the robustness of our findings against head motion, we verified the results using more stringent criterion. Subjects with excessive motion (i.e., translational or rotational motion greater than 2 mm or 2 °) were excluded from the subsequent analysis. All the selected participants (n = 124) were classified into child (17ASD/20TC), adolescent (28ASD/25TC) and adult (17ASD/17TC) groups. No significant differences of the age, gender, FIQ and mean FD were found between diagnostic groups within each age group. Two-way ANOVA was performed to ascertain main effects and interaction effects on whole brain ALFF maps. Gender, FIQ and mean FD were taken as covariates in the model. All the other preprocessing and processing methods remained the same as the main text. The ANOVA analysis demonstrated significant main effects of diagnosis in the right precuneus and bilateral cerebellum (Figure S3 A, B, C and D). Significant interaction effect was observed in the bilateral mPFC (Figure S3 E). These results highlight the crucial role of the default mode network in the development of ASD. This analysis confirmed the robustness of our findings to excessive head motion.

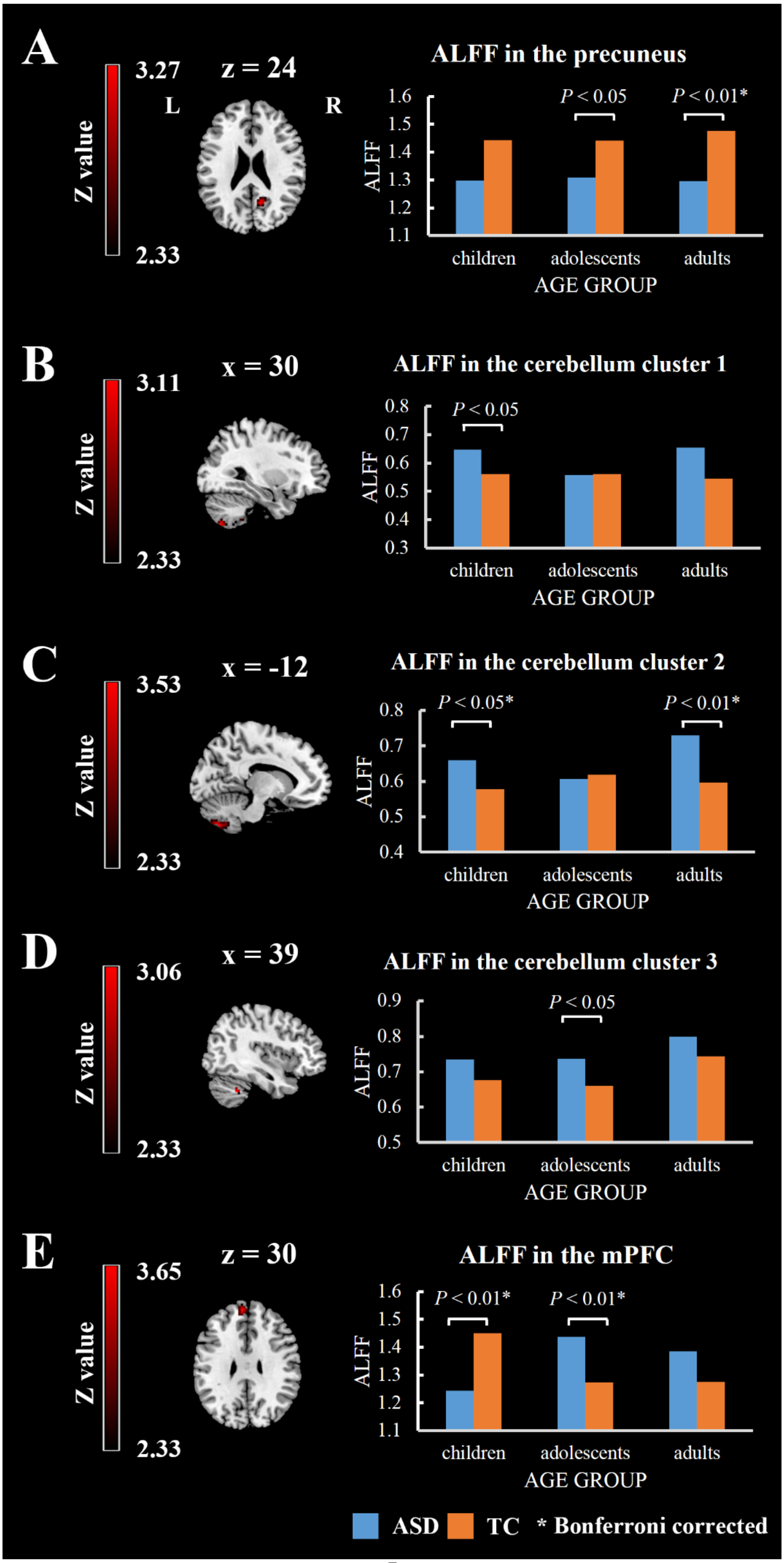

A
Z value
3.27
2.33
z = 24
L
R
ALFF in the precuneus
P < 0.05
P < 0.01*
ALFF
1.6
1.5
1.4
1.3
1.2
1.1
children
adolescents
adults
AGE GROUP
B
Z value
3.11
2.33
x = 30
ALFF in the cerebellum cluster 1
P < 0.05
ALFF
0.8
0.7
0.6
0.5
0.4
0.3
children
adolescents
adults
AGE GROUP
C
Z value
3.53
2.33
x = -12
ALFF in the cerebellum cluster 2
P < 0.05*
P < 0.01*
ALFF
0.8
0.7
0.6
0.5
0.4
children
adolescents
adults
AGE GROUP
D
Z value
3.06
2.33
x = 39
ALFF in the cerebellum cluster 3
P < 0.05
ALFF
0.9
0.8
0.7
0.6
0.5
children
adolescents
adults
AGE GROUP
E
Z value
3.65
2.33
z = 30
ALFF in the mPFC
P < 0.01*
P < 0.01*
ALFF
1.6
1.5
1.4
1.3
1.2
1.1
children
adolescents
adults
AGE GROUP
ASD
TC
* Bonferroni corrected

Figure S3 Significant diagnosis-related effects in replication analysis of head motion. Significant main effect of diagnosis on ALFF in the right precuneus (A) and cerebellum (B, C and D). Significant diagnosis-by-age interaction effect in the mPFC (E). mPFC: medial prefrontal cortex; ASD: autism spectrum disorder; TC: typical controls; * indicates Bonferroni corrected.

4. Additional analyses of FIQ and mean FD

Though the ANOVA analysis in primary analysis was adequately corrected for nuisance covariates, we conducted additional analyses to exclude the effects of FIQ and mean FD on diagnosis-by-age interaction effect. Two-way ANOVA analyses with diagnosis (two levels: ASD and TC) and age (three levels: child, adolescent and adult) as between-subject factors were performed using FIQ and mean FD, respectively. No significant interaction between diagnosis and age was observed in both analyses. In addition, Pearson correlation analyses were performed between ALFF values in the identified interaction cluster and FIQ, as well as mean FD. No significant correlation was found in the correlation analyses. These results suggest that the identified diagnosis-by-age interaction effect wasn't contributed by FIQ or mean FD.

5. Replication analysis using other datasets

To demonstrate the robustness of our results, primary findings were verified using other datasets. We examined the other ABIDE datasets and chose the dataset which contains most subjects at childhood, adolescence and adulthood, respectively. Stanford University (Stanford) (13 ASD and 11 TC), University of Michigan (UM) (23 ASD and 29 TC) and University of Utah School of Medicine (USM) (25 ASD and 29 TC) datasets were selected for child, adolescent and adult groups, respectively. Additional

analyses on the identified diagnosis-related clusters (bilateral mPFC, right precuneus and left middle occipital gyrus) were conducted. Two-sample *t*-test was performed to compare the strength of ALFF between ASD and TC groups within each age group. Results were similar to those emerging from primary analyses. Compared with age-matched control groups, children with ASD showed significant decreased ALFF in the mPFC and right precuneus, adolescents with ASD showed significant increased ALFF in the mPFC while adults with ASD showed significant decreased ALFF in the right precuneus. This analysis confirmed the robustness of our initial findings (Figure S4).

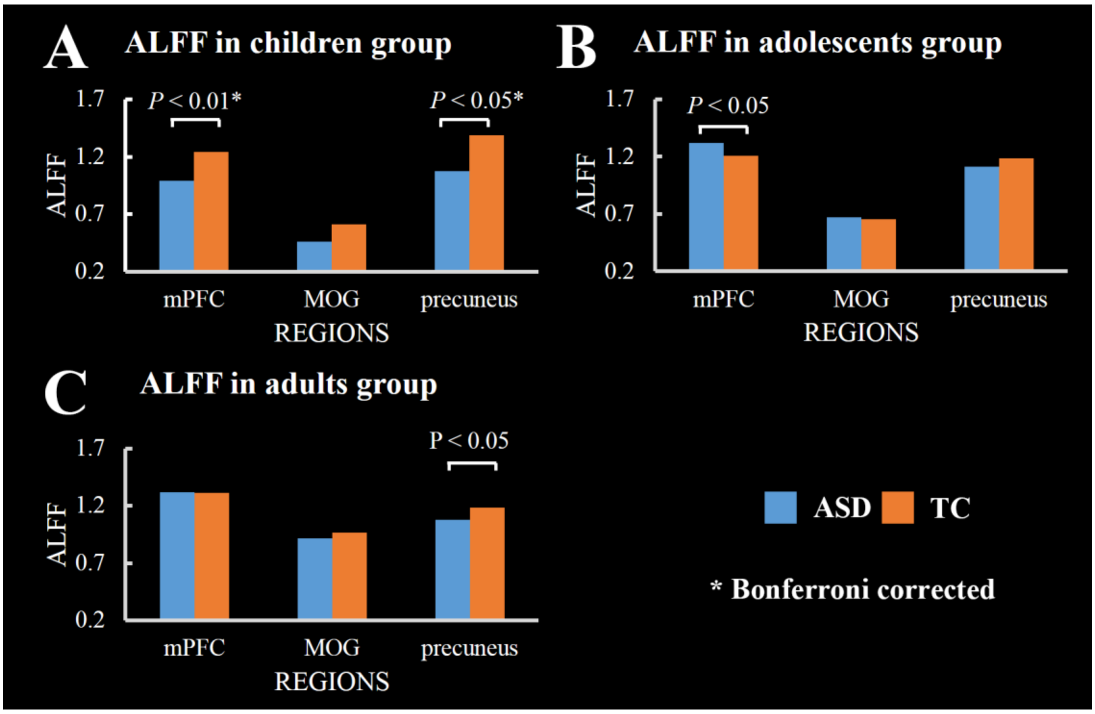

Figure S4 Replication analysis results using other datasets. (A) Children group results in Stanford dataset. (B) Adolescents group results in UM dataset. (C) Adults group results in USM datasets. mPFC: medial prefrontal cortex; MOG: Middle occipital gyrus; ASD: autism spectrum disorder; TC: typical controls; * indicates Bonferroni corrected.

6. Replication analysis of symptom severity prediction analysis

Since we didn’t include nuisance regressors (i.e., gender, FIQ and mean FD) in the

regression models in original analyses, we conducted additional multivariate regression analyses including gender, FIQ and mean FD in the regression models. Given that ALFF interacts with age during the development of ASD, age was also included as a factor in the regression model. For standardization, age, gender, FIQ and mean FD were divided by group mean value for each ASD subject, respectively. Multivariate support vector regression (SVR) approach was utilized to model the relationship between the dependent variable (communication, social, restricted and repetitive behaviors subscore of ADOS) and the multiple independent variables (voxel-wise ALFF values in brain regions showing significant main effects of diagnosis and interaction effects and standardized age, gender, FIQ and mean FD). All the other data processing method remained the same as primary analyses in the main text. Overall, results were consistent with our primary analysis. ALFF in the mPFC, right precuneus and left middle occipital gyrus together with age, gender, FIQ and mean FD predicted the social subscore of ADOS in the ASD group (p = 0.006, Bonferroni corrected). However, regression analysis predicting scores on communication or restricted and repetitive behaviors domains of ADOS didn't yield significant correlations.

7. Regression analyses with age as a continuous regressor

We also conducted whole-brain analyses exploring linear as well as quadratic relationships with age as a continuous regressor to see whether it similarly identifies the diagnosis-by-age interaction in the mPFC. To do so, we performed voxel-based multiple regressions with voxel-wise ALFF values as dependent variable and age as a continuous regressor of interest for ASD and TC groups, respectively. Gender, FIQ and

mean FD were taken as nuisance regressors in the regression model. Gaussian random field theory was employed for multiple comparisons correction (voxel-level $p < 0.05$, cluster-level $p < 0.05$). Quadratic results showed a significant positive quadratic relationship between age and ALFF values in the mPFC in TC group (U-shaped), while we didn't identified any mPFC cluster in quadratic effects of ASD group. Additionally, we performed two-way ANOVA analysis with diagnosis (two levels: ASD and TC) and age as between-subject factors on ALFF values in the mPFC cluster found in TC group. Significant interaction effect was observed for ALFF in the mPFC cluster ($p < 0.05$). In linear regression analysis, both ASD and TC group showed increased ALFF with increasing age in several mPFC clusters. However, we also found a cluster in the mPFC where TC group showed a negative linear relationship between age and ALFF values, while ASD group showed no negative relationship in the mPFC. Additional ANOVA analysis observed significant diagnosis-by-age interaction effect in the mPFC cluster identified negative relationship in TC group ($p < 0.01$). No significant interaction effect was found in mPFC clusters which exhibited positive relationship in ASD or TC group. These findings demonstrated that both linear and quadratic analyses similarly identified significant diagnosis-by-age interaction effects in the mPFC as our primary analysis (Figure S5).

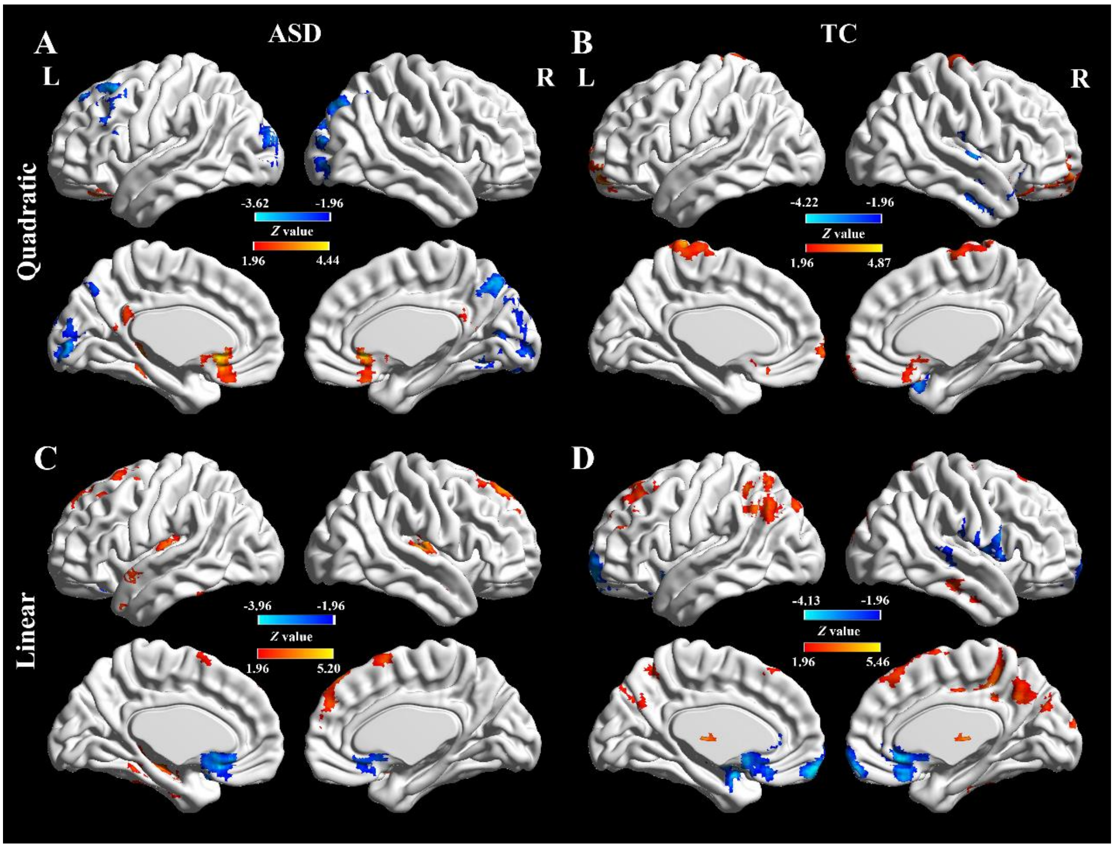

Figure S5 Regression analyses with age as a continuous regressor. Quadratic relationships between ALFF and age in ASD (A) and TC groups (B). Linear relationships between ALFF and age in ASD (C) and TC groups (D).

References

1 Lai, M. C. *et al.* Biological sex affects the neurobiology of autism. *Brain : a journal of neurology* **136**, 2799-2815 (2013).

2 Lai, M. C. *et al.* Cognition in males and females with autism: similarities and differences. *PloS one* **7**, 440-440 (2012).

3 Schaer, M., Kochalka, J., Padmanabhan, A., Supekar, K. & Menon, V. Sex differences in cortical volume and gyrification in autism. *Molecular Autism* **6**, 1-14 (2015).
